# Supplementary material for: Analysis of the interval between submission and publication in genetics journals
Source: PLoS One. 2023 May 17;18(5):e0284866. doi: 10.1371/journal.pone.0284866 (PMC10191348; doi:10.1371/journal.pone.0284866)
Supplement: Attachment — Submitted filename: Response to Reviewers.docx [file pone.0284866.s001.docx]

Response to Reviewers

Dear Dr. Chenette,

We thank you and the reviewers for your time in reviewing our paper and providing valuable comments. We have carefully considered the comments and provide below a point-by-point response to address each and every one of them. We hope that the manuscript, after careful revisions, will meet your high standards. The authors welcome further constructive comments if any.

All modifications in the manuscript have been highlighted in yellow.

Sincerely,

Rafael Leal Zimmer, MSc

PhD student,

Universidade Federal do Rio Grande do Sul

**Reviewer #1:**

Dear authors,

Thanks for letting me read and review your article.

Although your article has many exciting features, it demands the publication of some necessary corrections.

First, and referring to the literature review and problem-oriented introduction, there needs to be more problematization about productivity, availability of reviewers, and other connected factors that should be presented in this section of your paper. For example, some work had been reflected in the productivity of the peer review process. Articles like "Teixeira da Silva, J. A., & Dobránszki, J. (2015). Problems with traditional science publishing and finding a wider niche for post-publication peer review. Accountability in research, 22(1), 22-40." could serve to produce a more detailed and theory-driven literature review for your analysis and later discussion.

**Response**: Thank you very much for this comment. We have adjusted the introduction and discussion according to your suggestions.

Later on, when the paper goes to the results and discussion, the article uses several studies to compare the timing from submission to publication. These studies deserve their own space in a literature or problem-oriented literature section. To use such work in your results and discussion appears not grounded in the line of argument of your article.

**Response**: Thank you very much for this comment. We have removed the studies/references from the results section and adjusted the discussion as mentioned previously.

Finally, when the paper goes into the crucial discussion about the article by continents, authors refrain from getting involved with essential discussions in knowledge production that happened in the last years—for example, center-periphery in knowledge production and global knowledge circulation phenomena. If the paper wants to discuss such topics, it is necessary to review the literature about it and confront it in this section of the article.

**Response**: Thank you for your suggestion, however we feel that this is beyond the scope of the present article. The main objective is to first describe the distribution of production in the continents for each quartile and bring attention to the problem. On the other hand, a more profound study of the determinants of such results is certainly warranted and should include the aspects mentioned in your comment, such as the recently observed center-periphery phenomenon as well as other current scenarios such as, for instance, evident differences between covid vs. non-covid related publications . We have mentioned this aspect among the limitations of the study.

Finally, you mention, "Our results may contribute in the development of strategies to expedite the process of scientific publishing in the field, and to promote equity in knowledge production and dissemination for researchers from all continents." Developing strategies to promote equity in knowledge production is an important issue that connects with your results that you could have expanded in a better sense. Therefore, such strategy developments deserve at least a few paragraphs in your conclusions and policy recommendations. All in all, your paper presents a critical analysis that demands more engagement with the literature and, consequently, to improve your article's discussion and conclusions.

Response: Thank you very much for this comment. We have adjusted the conclusions according to your suggestions.

**Reviewer #2:** The present research intends to show an important part of scientific productivity such as publication time. In order for the work not to be considered as a simple and not very detailed description, it should have an adequate statistical rigor, showing the work of cleaning the databases, what criteria are considered for the elimination of missing or lost data, protocols of the tradition and current paradigms of scientometrics and an exhaustive review of the literature to consider which are the statistical methodologies most used for this purpose. In that sense, this article precisely misses these points. Despite its weakness, it is recommended to enhance its analysis with more advanced statistics for its analysis. There are countless laws from scientometrics that could help to pose more challenging questions with deeper and richer analysis. The analysis of quartiles is interesting but only as a descriptor factor; however, the real problem is in the limited challenge of the research

**Response**: Thank you very much for your comments. Regarding cleaning of databases, it was performed in two steps. First, journals that do not provide submission (S), acceptance (A), and publication (P) dates were excluded from the sample, with a new one drawn from the original pool. For manuscripts not presenting all dates, the interval was calculated only for the available dates (e.g. SP only). Therefore, we have slightly different numbers in each interval, as presented in the footnote of table 3. We added a comment on the study proposed by Shelton (2020) relating Lotka's Law and Bradford's Law to national funding, and suggested that the time frame chosen for analysis (e.g. 1 year after funding) may bias the results towards higher impact journals, in which some countries are under-represented.

**Reviewer #3**: This is a well written and innovative paper. The authors developed a very original idea and use a interesting methodology to study it. The conclusions are relevant and this paper may be very useful to PlosOne readers.

**Response**: Thank you very much for your comments.
